# Gene Expression Analysis of the Stress Response to Lithium, Nickel, and Zinc in *Paracentrotus lividus* Embryos

**DOI:** 10.3390/toxics10060325

**Published:** 2022-06-14

**Authors:** Rosa Bonaventura, Caterina Costa, Irene Deidda, Francesca Zito, Roberta Russo

**Affiliations:** Istituto per la Ricerca e l’Innovazione Biomedica, Consiglio Nazionale delle Ricerche, Via Ugo La Malfa 153, 90146 Palermo, Italy; asg.eri@outlook.it (C.C.); irene.deidda@irib.cnr.it (I.D.); francesca.zito@irib.cnr.it (F.Z.)

**Keywords:** echinoderm, pollution, invertebrates, stress response, metals, transcripts

## Abstract

Many anthropogenic pollutants such as metals are discharged into the marine environment through modern sources. Among these, lithium (Li), nickel (Ni), and zinc (Zn) can interfere with biological processes in many organisms when their concentration rises. These metals are toxic to sea urchin embryos, affecting their development. Indeed, animal/vegetal and dorso/ventral embryonic axes are differently perturbed: Li is a vegetalizing agent, Ni can disrupt dorso-ventral axis, Zn can be animalizing. To address the molecular response adopted by embryos to cope with these metals or involved in the gene networks regulating embryogenesis, and to detect new biomarkers for evaluating hazards in polluted environments in a well-known *in vivo* model, we applied a high-throughput screening approach to sea urchin embryos. After fertilization, *Paracentrotus lividus* embryos were exposed to Li, Ni, and Zn for 24/48 h. At both endpoints, RNAs were analyzed by NanoString nCounter technology. By in silico analyses, we selected a panel of 127 transcripts encoding for regulatory and structural proteins, ranked in categories: Apoptosis, Defense, Immune, Nervous, Development, and Biomineralization. The data analysis highlighted the dysregulation of many genes in a metal-dependent manner. A functional annotation analysis was performed by the KEEG Orthology database. This study provides a platform for research on metals biomarkers in sea urchins.

## 1. Introduction

The use of metals has accompanied civilization and is closely linked to anthropogenic activities due to their chemical characteristics [1]. Metals are present in many environments at variable concentrations. Some of them are essential, for example, zinc (Zn), copper, and manganese, which play key roles in many cellular metabolic processes in living organisms, while other metals are non-essential, not having a physiological role, as for example lead, cadmium (Cd), and lithium (Li) [1,2]. Others, such as nickel (Ni), are essential elements in plants and microorganisms, while in animals their essentiality is controversial [3]. Metals can be released into the environment by different types of human activities such as mining, urban runoff, sewage discharge, and chemical compounds applied to crops for insect or disease control [2]. Regardless of their essentiality or not, when metal concentrations raise, they become chronic and toxic with harmful effects for both marine and terrestrial organisms, including humans [2]. In this regard, several metals have been included in the 2019 Substance Priority List by the Agency for Toxic Substances and Disease Registry (ATSDR) (https://www.atsdr.cdc.gov/spl/ (accessed on 30 June 2021)), because they pose the most significant potential threat to human health due to their known or suspected toxicity. The potential hazards for human exposure, and the bioavailability of some metals, such as lead, cadmium, zinc, copper, manganese, iron, mercury, arsenic, and barium, have been described in marine environments, as well as metals bioaccumulation by numerous marine organisms [4,5]. Although there are many studies on the toxic effects and accumulation of metals in different marine organisms [5], relatively little is known about their molecular effects. This is also the case with Ni, Zn, and Li. Ni and Zn are essential elements for the normal growth and development of aquatic organisms if present in trace amounts [6]. In seawater, Ni and Zn are normally present at low concentrations, 0.1–1.0 and 0.006–0.12 μg L^−1^, respectively [6]. However, at high concentrations or in chronic conditions, they can have adverse effects on many marine biota [3,6]. Concentrations of up to 250 μg/L of dissolved Ni can be detected in coastal areas, and up to 2 mg/L can be found in industrially contaminated water [3]. Ni toxicity has been studied in different marine species that showed diverse sensitivity to different Ni concentrations [3,7]; Ni EC10s values ranged from 2.9 μg/L for the embryos of the tropical sea urchin *Diadema antillarum* to 20,760 μg/L for the larvae of the sheepshead minnow *Cyprinodon variegatus* [7]. Zn level depends on the geological regions and pollution sources (air, soil, and rivers), with seawater salinity and pH affecting the relative proportion of different inorganic complexes with uncomplexed Zn^2+^, which is the most bioavailable form of Zn at the pH of seawater [6]. For example, among the marine organisms (crabs, prawns, and six different varieties of fish species) collected in an anthropic impacted area near Mumbai city (India), the highest concentration of Zn (31.2 mg/kg) was found in crabs [8]. Although from a geochemical point of view Li is a rare metal, nowadays many electronic devices in daily use require lithium-ion batteries, thus Li will increasingly be a key resource in the future [9]. Some studies recorded the highest levels of Li in natural waters in Chile and Bolivia [9] and rivers in China [10], evaluating its effects on the relative impacted population. Biochemical and molecular effects of Li on signal transduction cascades, hormonal and neural regulation, ion transport, and gene expression were already known [11]. Moreover, Li is one of the most effective drugs used in medicine to cure mental illnesses [12]. Minor attention has been given to Li as a pollutant agent in seawater. Very recently, a large-scale survey of Li concentrations was conducted in 33 species of bivalves, cephalopods, crustaceans, and fishes (whole organisms and different organs) from three distinct geographic areas [13]. The study showed that Li is homogeneously distributed in the ocean (at 0.18 μg/mL), while its concentrations in soft tissues vary greatly (from 0.01 to 1.20 μg/g dry weight) [13]. 

The sea urchin embryo is one of the best-studied model organisms since the birth of experimental embryology. It was classically used in experiments on blastomere isolation and recombination [14]. More recently, the sea urchin embryo was used to study the mechanisms of gene regulation in early development [15] and to describe the sequential regulatory changes that specify and diversify the embryonic cells [16]. In this respect, Li, Ni, and Zn have been useful to study the gene regulatory networks (GRNs) in sea urchin embryos, i.e., they have been considered models to describe the specification of the endo-mesoderm and the ectoderm [17,18,19], exploiting their effects on the animal/vegetal (A/V) and dorso-ventral (D/V) axes establishment. Li is reported to be a vegetalizing agent, inducing presumptive ectoderm to differentiate as endo-mesoderm, sometimes producing exogastrulae with an everted gut [20]. Ni disrupts the D/V axis, forming radialized embryos, mainly affecting ectodermal differentiation and pattern formation of mesenchyme cells, overexpressing ventral genes at the expense of the dorsal ones [21]. Sub-lethal concentrations of Zn induce animalization, i.e., increase the specification of the ectoderm cells at the animal pole and reduce the specification of endo-mesoderm territories [19]. 

The sea urchin embryo has also been used successfully as a model to analyze the molecular defense systems adopted to cope with a variety of pollutants, including metals [22]. Furthermore, high-throughput screening approaches are increasingly used to analyze the molecular response to chemicals in marine invertebrates. For instance, transcriptomic analysis was applied to evaluate crude oil effects on the oyster *Crassostrea virginica* [23]. Different approaches have been used to identify differentially expressed genes in *Strongylocentrotus nudus* embryos exposed to triclosan, i.e., cDNA microarray [24], and to characterize the response of *Paracentrotus lividus* embryos to UV radiation, cadmium, and their combination, i.e., NanoString nCounter technology [25].

The aim of this study was to obtain an overview of the genes modulated in *P. Lividus* sea urchin embryos to cope with acute doses of LiCl, NiCl_2,_ and ZnSO_4_ after 24 and 48 h of treatments, by the multiplex analysis of NanoString nCounter technology. In total, we selected 127 genes organized into six categories, i.e., Apoptosis (*Ap*), Defense (*Def*), Immune (*Imm*), Nervous (*Ner*), Development (*Dev*), and Biomineralization (*Bm*), and took into consideration the territorial expression and functions of the analyzed genes. In addition, we tried to identify the potential biomarker genes particularly sensitive to the three metals, i.e., Li, Ni, and Zn, respectively, and greatly affected by them.

## 2. Materials and Methods

### 2.1. Embryo Culture

Gametes were collected from gonads of the *P. lividus* sea urchin harvested along the northwestern coast of Sicily. Eggs were fertilized and embryos were reared at 18 °C in Millipore (Billerica, MA, USA) filtered seawater (MFSW) containing antibiotics (50 mg/L streptomycin sulfate and 30 mg/L penicillin).

### 2.2. Metals Treatment of P. lividus Embryos 

Caution: LiCl, NiCl_2_ and ZnSO_4_ are hazardous chemicals and should be handled carefully.

To study the embryonic response induced by Li, Ni, and Zn in *P. lividus* sea urchin embryos, we took advantage of a previous study [26]. Briefly, embryos were continuously cultured in the presence of 30 mM LiCl (Li) (Sigma-Aldrich, St. Louis, MO, USA), 0.5 mM NiCl_2_ (Ni) (Sigma-Aldrich, St. Louis, MO, USA) and 0.1 mM ZnSO_4_ (Zn) (Sigma-Aldrich, St. Louis, MO, USA), henceforth defined as Li-embryos, Ni-embryos, and Zn-embryos, respectively. Metals were added to embryo cultures 30 min. after fertilization. To collect embryos for subsequent analyses, we chose the endpoints corresponding to 24 and 48 h post-fertilization (hpf), when controls (unexposed embryos) reached gastrula and pluteus stages, respectively. Embryo morphologies were monitored using a microscope Axioscop 2 plus (Zeiss, Jena, Germany) both on live and fixed embryos with 0.1% formaldehyde in seawater. Images were recorded by an Axiocam camera (Model 412–312, Zeiss, Jena, Germany).

### 2.3. Indirect Immunofluorescence (IF)

IF experiments on whole-mount embryos were performed as described previously [27] with some modifications. Briefly, paraformaldehyde (PFA, Sigma-Aldrich, St. Louis, MO, USA)-fixed embryos were incubated with TBST [10 mM Tris-HCl (pH 8), 150 mM NaCl, 0.1% Tween 20] containing 4% donkey serum (DS), and 0.5% BSA (DS/BSA-TBST) for 1 h. 

The ciliary band was specifically labeled with a monoclonal antibody 295 (kindly provided by Prof. McClay, Department of Biology, Duke University, 124 Science Drive, Box 90338, Durham, NC 27708, USA) diluted 1:5 in TBST. Rabbit polyclonal anti-Sox2 (Sigma-Aldrich, St. Louis, MO, USA) was used at a dilution of 1:25 in 0.25% DS-TBST. Both primary antibodies were incubated overnight at 4 °C with specimens and, after washing in TBST, the fluorescent-labeled secondary and appropriate antibodies (Alexa Fluor 488-conjugated goat anti-mouse or Alexa Fluor 555-conjugated donkey anti-rabbit) (Invitrogen Molecular Probes, Carlsbad, CA, USA) were diluted 1:200 in TBST and incubated for 1 h at room temperature. 

For double-labeling of 295 and Sox2, embryos were incubated in blocking buffer DS/BSA-TBST for 1 h and incubated with both primary antibodies (diluted 1:5 for 295 and 1:25 for Sox2) in 0.25% DS-TBST overnight at 4 °C. Secondary antibodies were used as described in the single immunostaining. 

All embryos were observed under a Leica DM4000 microscope (Leica Microsystems, Wetzlar, Germany), equipped for epifluorescence, and images were recorded using a digital camera system. Images were edited using Adobe Photoshop CS2 software. Negative controls were performed for every set of experiments by omitting the primary antibodies.

### 2.4. nCounter NanoString Gene Expression Assay

Embryos from the samples treated with Li, Ni, and Zn, respectively, were processed following the manufacturer’s instructions of the RNeasy mini-Kit (Qiagen, Germantown, MD, USA) to extract total RNA, then quantified using the D30 bio-photometer (Eppendorf, Hamburg, Germany). 100 ng of total RNA was used to apply nCounter NanoString technology (https://www.nanostring.com/company/about-us (accessed on 1 January 2018)) provided by Diatech Labline SRL (Jesi, Italy), in order to analyze the expression of a panel of 127 *P. lividus* transcripts, selected and retrieved from the National Centre for Biotechnology Information (NCBI) nucleotide database (http://NCBI.nlm.nih.gov (accessed on 1 January 2018)). Briefly, unique custom-made probes developed for the selected *P. lividus* sequences were hybridized with total RNA samples. Then, each hybridized barcoded transcript was detected and counted by a digital analyzer for image acquisition and data recording. The expression level was measured after normalization of the resulting digital counts of each transcript with those of the *Pl-Z12-1* reference gene (Accession Number: LT900344) [28]. Then, the normalized counts of each transcript were compared between control and treated embryos to obtain the fold-change values.

We organized the analyzed genes into six arbitrary categories, in alphabetical order: Apoptosis (*Ap*), Biomineralization (*Bm*), Defense (*Def*), Development (*Dev*), Immune (*Imm*), and Nervous (*Ner*). The heat map was generated by applying the conditional formatting in Microsoft Excel, setting as rule three range of fold values, <−2 (violet), >+2 (green), and between −2 and +2 (yellow) corresponding to the colors indicated in brackets.

### 2.5. Real-Time Quantitative PCR (qPCR) 

Total RNA from *P. lividus* control and treated embryos, the same utilized for the nCounter Nanostring analysis, was reverse transcribed to obtain the corresponding cDNA as described in Russo et al. [26]. In order to quantify gene expression, the cDNAs were amplified by using SYBR Green technology, based on a Comparative Threshold Cycle Method [29], according to the manufacturer’s instructions (Applied Biosystems StepOnePlus instrument, Grand Island, NY, USA) and as previously described [26]. *Pl-Z12-1* mRNA was used as a reference gene. The primer sequences of the genes utilized for qPCR were previously reported: *14-3-3* [30], *metallothionein* (*mt*) [31], *jun* [32], *alx1*, *carbonic anhydrase (can)*, *galectin-8 (gal-8)* [25]. The primer specificity and accuracy were confirmed by the “melting curve”, carried out during the real-time PCR.

### 2.6. KEGG Enrichment Analysis

Enrichment analysis was performed for all the genes modulated by each metal and those modulated by all the three metals, using the integrated KEGG Orthology (KO) database (in particular, KEGG pathway map and BRITE hierarchy databases; https://www.genome.jp/kegg/ko.html (accessed on 1 June 2022)), selecting *S. purpuratus* as an organism (entry code: spu; genome number: T01019). The KO database includes molecular functions that are represented in terms of functional orthologs. Each gene is given a KO identifier, which represents a functional ortholog defined on the basis of the similarity of its sequence with orthology genes from other organisms, to generate organism-specific versions of KEGG pathways (database, path) and BRITE hierarchies (database, br), to deduce high-level functions of the organism. 

### 2.7. Statistical Analysis 

QPCR values are reported as the mean of three independent qPCR analyses ± SD, using cDNAs obtained by the same experiment of *P. lividus* embryos exposed to Li, Ni, and Zn used for the nCounter NanoString analysis. QPCR decimal values were transformed into negative values to compare them to NanoString values. Results were compared using OneWay analysis of variance, ANOVA followed by Tukey’s HSD test, used as a post-hoc test for mean comparison. All the analyses were performed using the OriginPro 8.1 statistical program (OriginLab Corp), and the level of significance was set to *p* ≤ 0.05.

## 3. Results

### 3.1. Developmental Effects of Li, Ni and Zn on Sea Urchin Embryos

It is known that metals such as Li, Ni, and Zn perturb territorial differentiation during the development of diverse sea urchin species. We have previously shown that the sublethal doses of 30 mM LiCl, 0.5 mM NiCl_2,_ and 0.1 mM ZnSO_4_ produced homogeneous populations of abnormal *P. lividus* embryos, each with a characteristic morphology [26,33]. Here, we selected the same doses to continuously treat the embryos for 24 and 48 hpf for subsequent molecular analyses. 

**Figure 1:**
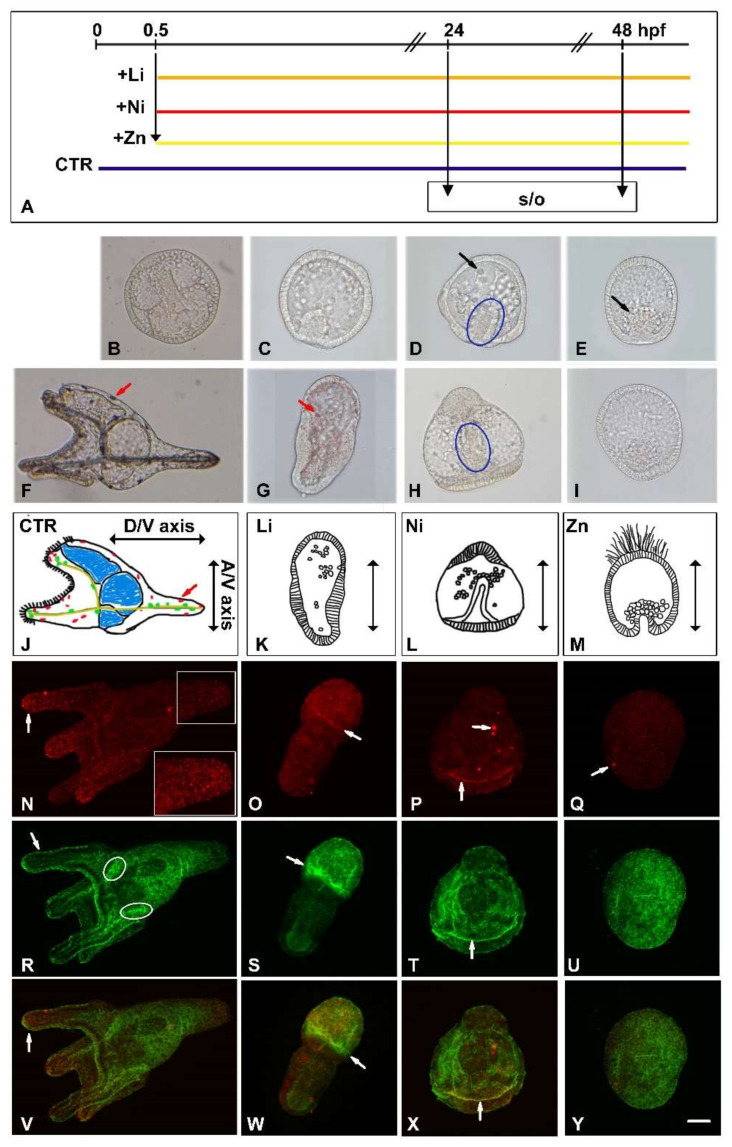
Morphologies of *P. lividus* embryos treated with Li, Ni, and Zn. (**A**) Schematic drawing of Li, Ni and Zn treatment experiments. Black line, hpf, in the upper part; the first vertical arrow indicates addition of 30 mM LiCl (Li), 0.5mM NiCl_2_ (Ni), 0.1mM ZnSO_4_ (Zn) at 0.5 hpf. Periods of treatment with Li, orange line; Ni, red line; Zn, yellow line. Untreated embryos (CTR), blue line. s/o indicates sampling (s) for NanoString nCounter gene expression assay and qPCR analyses and (o) microscopic observation. Embryos were observed at 24 (**B**–**E**) and 48 (**F**–**I**) hours post-fertilization (hpf). Untreated embryos: (**B**) control gastrula; (**F**) control pluteus. (**C**,**G**) Li-embryos; (**D**,**H**) Ni-embryos; (**E**,**I**) Zn-embryos. Blue ellipses in (**D**,**H**) indicate short archenteron. Black arrows in (**D**,**E**) indicate cells grouped inside the blastocoelic cavity. Red arrows in (**F**,**G**) indicate pigment cells. Schemes of embryos at 48 hpf: control pluteus, CTR (**J**); Li-embryo, Li (**K**); Ni-embryo, Ni (**L**); Zn-embryo, Zn (**M**). Animal/Vegetal (A/V), Dorso/Ventral (D/V) axes are indicated in the scheme of pluteus embryo. (**N**–**Q**) Anti-Sox2 antibody (red); (**R**–**U**) Ab-295 (green); (**V**–**Y**) Merged images (yellow) of the relative Anti-Sox2 (**N**–**Q**) and Ab-295 (**R**–**U**) images, respectively. The image in the white frame in (**N**) indicates 1.4× magnification of the pluteus apex, where brightness and contrast have been increased. White arrows in (**N**–**Q**) indicate Sox2-positive cells. White ellipses in (**R**) indicate small central ganglionic structures. White arrows in (**R**–**T**) and (**V**–**X**) indicate ciliary band. Bar 20 μm for (**B**–**I**) and (**N**–**Y**).

Figure 1A shows the experimental design and Figure 1B–I confirms the previously observed morphologies and therefore the reproducibility of the experiment. In particular, at 24 hpf, the untreated normal embryos were at the gastrula stage with an elongated archenteron and two triradiate spicules (corresponding to the initial formation of the skeleton) (Figure 1B). The vegetalized Li-embryo (reduced ectoderm and increased endomesoderm, see Ghiglione et al. [20]) had an early gastrula shape with a very short invaginated archenteron and many cells spread inside the blastocoelic cavity (Figure 1C). The radialized Ni-embryo also had a short archenteron (indicated by a blue ellipse in Figure 1D) with cells grouped inside the blastocoelic cavity, some of which were above the equatorial plane (black arrow in Figure 1D). The animalized Zn-embryo looked like a blastula with a group of cells arranged as a presumptive invaginating archenteron (black arrow in Figure 1E). At 48 hpf, untreated embryos reached the pluteus stage (Figure 1F, lateral view), with a tripartite gut, elongated and patterned spicules, and pigment cells (red arrow), see also the drawing in Figure 1J. At this stage, all treatments greatly affected embryos development, as shown by images (Figure 1G–I) and drawings (Figure 1K–M) reporting the different embryonic morphologies and the direction of the developmental axes, i.e., animal-vegetal (A/V) and dorso-ventral (D/V). Li-embryos had a typical endoderm-derived everted gut and a sphere with many pigment cells (red arrow) (Figure 1G,K). Ni-embryos had a straight gut, a thickened animal cap that provided a typical bell shape, and some cells grouped near the archenteron (blue ellipse in Figure 1H,L). Zn-embryos maintained a blastula-like shape (Figure 1I,M). To highlight the morphological diversities, we performed an indirect immunofluorescence (IF) analysis using the anti-Sox2 antibody (Figure 1N–Q), a marker of the nervous system [34], and 295 monoclonal antibody (Ab-295), which recognizes the epithelium of ciliated cells of the embryonic arms, surrounding the mouth, called ciliary band associated to the embryonic nervous system (Figure 1R–U) [35]. The anti-Sox2 is a commercial antibody against a synthetic peptide corresponding to residues 32–47 of human Sox2, homologous to the *Strongylocentrotus purpuratus* SoxB1. In normal embryos, anti-Sox2 labeled the ciliary band (see white arrow in Figure 1N) and some cells in the pluteus apex (see 1.4× magnification within the white frame). In metals-treated embryos, anti-Sox2 labeled a belt of cells in Li and Ni embryos, and very few cells in the Zn-embryos (see white arrows in Figure 1O–Q). In normal embryos, in addition to recognizing the ciliary band (Figure 1R, white arrow), the Ab-295 also labeled two small central ganglionic structures, indicated by white ellipses in Figure 1R. In Li-embryos, the Ab-295 clearly labeled the animal ectoderm separated from vegetal endomesoderm (Figure 1S). In Ni-embryos, the Ab-295 labeled the ciliary band delimited from the vegetal region (Figure 1T, white arrow), while in Zn-embryos, it labeled the embryo uniformly (Figure 1U). In the merged images (Figure 1V–Y), the yellow color highlights the co-localization of anti-Sox2 and Ab-295 in some cells of the ciliary band in normal embryos (Figure 1V, arrow) as well as in Li- and Ni-embryos (see arrows in Figure 1W and 1X, respectively).

### 3.2. Profiling Transcriptional Outputs in Li, Ni, Zn Treated Embryos

We applied a high-throughput molecular approach to analyze the gene expression profile of sea urchin embryos exposed to acute doses of Li, Ni, and Zn, with the aim of focusing on those genes differentially expressed to cope with these agents singly applied. 127 transcripts of the sea urchin *P. lividus* were specifically selected after a careful study from the NCBI database and used to perform the multiplex RNA analysis by nCounter NanoString technology in embryos at 24 and 48 hpf.

We chose to organize the selected genes into six categories described in Section 2. For some of them, we also reported some subcategories and their territorial expression, taking into consideration their functions (see Table S1).

**Figure 2:**
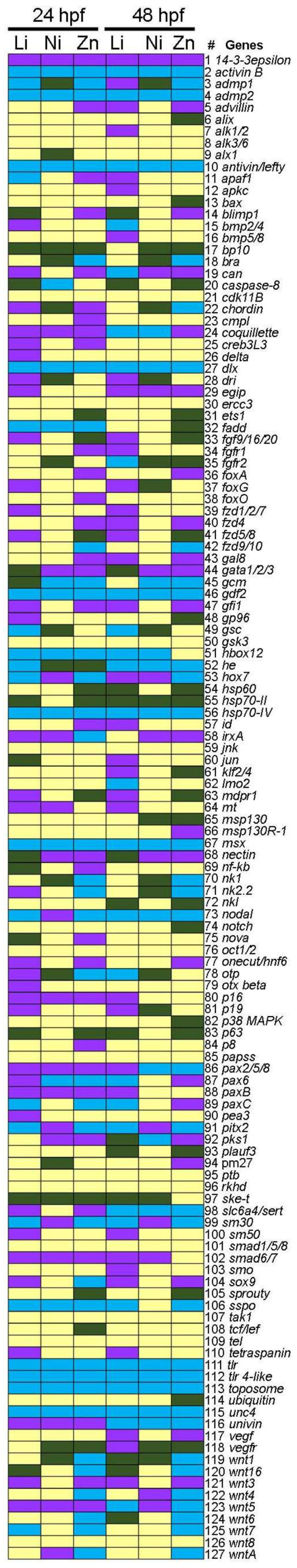
Heat map of the 127 genes analyzed following metal treatments. The heat map was generated using conditional formatting on Microsoft Excel. Genes are reported in alphabetical order. Violet, green, and light yellow indicate fold values <−2, >+2 and between −2 and +2, respectively; light blue indicates not quantifiable genes. Li, embryos treated with 30 mM LiCl; Ni, embryos treated with 0.5 mM NiCl_2_; Zn, embryos treated with 0.1 mM ZnSO_4_. Hpf, hours post-fertilization.

In Figure 2, the heat map shows an overview of the genes modulated by the treatments with the three different metals, where fold-change values higher or lower than +2 and −2 (compared to controls) were considered as significant increase/decrease, as suggested by the NanoString technique. The fold-change expression values of the 127 genes, calculated on previously normalized data (see M and M), are reported in Table S1. The cell fill color identifies the responsive genes, i.e., those upregulated in green (>+2), downregulated ones in violet (<−2), and not responsive ones in light yellow. The light blue identifies the no-quantifiable genes (digital counts lower than 50), assuming that they were very poorly expressed in the control and/or in the samples and thus to be excluded according to the manufacturer’s instructions. 

**Figure 3:**
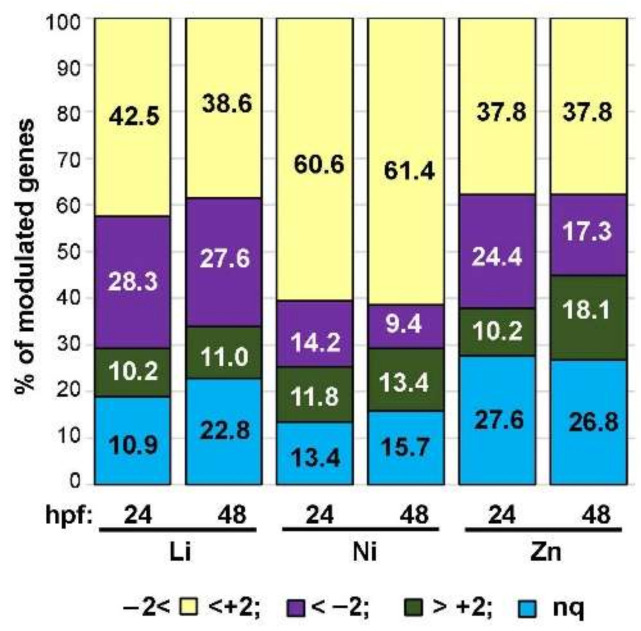
Percentages of genes modulated by metal treatments. Percentages of genes modulated by Li (30 mM LiCl), Ni (0.5 mM NiCl_2_) and Zn (0.1 mM ZnSO_4_) treatments at 24 and 48 hpf (hours post-fertilization). The fill colors indicate the upregulated genes in green (>+2), downregulated genes in violet (<−2), not responsive in light yellow (between −2 and +2), and not quantifiable (nq) in light blue.

Figure 3 summarizes the percentages of genes modulated or not by the metals at 24 and 48 hpf using the color code previously established for the heat map. In general, most of the genes were not affected (values between −2 and +2, light yellow in Figure 3) by metal treatments at both endpoints analyzed or were not quantified (light blue in Figure 3). Notably, the highest percentages of unaffected genes (60.6% and 61.4% at 24 and 48 hpf, respectively) were observed in Ni-embryos. As for the affected genes, although the three metals modulated the analyzed genes differently, these modulations did not substantially vary at the two endpoints of 24 and 48 hpf for each metal. In particular, Li triggered the modulation of 38.6% of the genes (49 in total) at both 24 and 48 hpf, with higher values for the downregulated genes (28.3% and 27.6%) than for the upregulated ones (10.2% and 11%) at both endpoints. As already highlighted, Ni treatment modulated a low percentage of genes, i.e., 26% (33 out 127 genes) at 24 hpf and 22.8% (29 out 127 genes) at 48 hpf, with similar percentages between downregulated (14.2% and 9.4%) and upregulated (11.8% and 13.4%) genes at both endpoints. Even in Zn-embryos, similar percentages of modulated genes were observed at both endpoints, i.e., 34.6% (44 genes) at 24 hpf and 35% (45 genes) at 48 hpf, with noteworthy differences between up- (24.4% vs. 17.3%) and downregulated (10.2% vs. 18.1%) genes (Figure 3).

**Figure 4:**
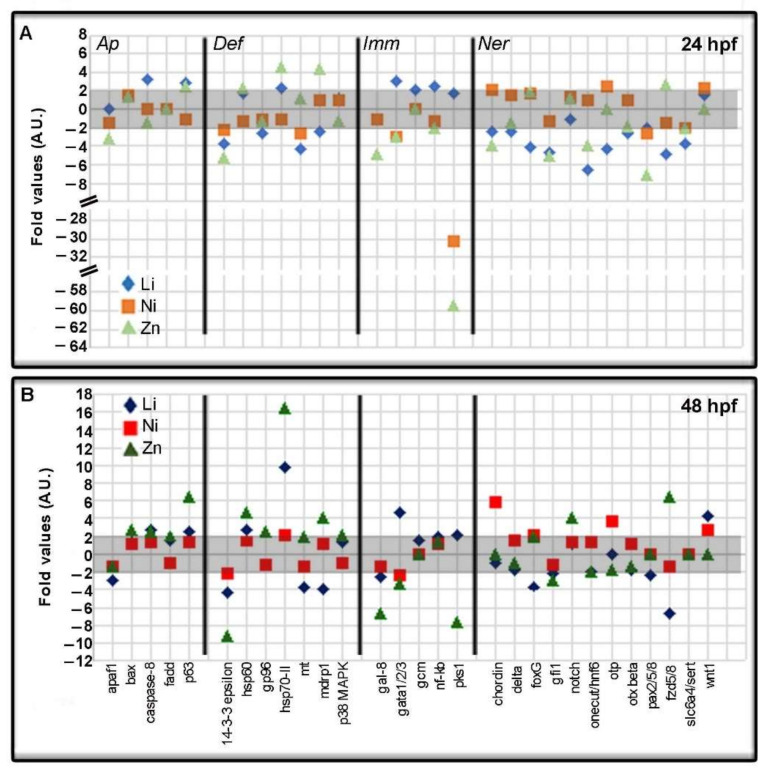
Apoptosis, Defense, Immune, and Nervous genes are modulated by Li, Ni, and Zn treatments. The grey boxes indicate the threshold for insignificant changes following Li, Ni, and Zn treatments, i.e., fold values between −2 and + 2 at 24 (**A**) and 48 (**B**) hours post-fertilization (hpf). Symbols in the plot at 24 hpf (**A**): light blue rhombus, Li-embryos; orange square, Ni-embryos; light green triangle, Zn-embryos. Symbols in the plot at 48 hpf (**B**): blue rhombus, Li-embryos; red square Ni-embryos; green triangle, Zn-embryos. *Ap*, Apoptosis genes; *Def*, Defense genes; *Imm*, Immune genes; *Ner*, Nervous genes.

**Figure 5:**
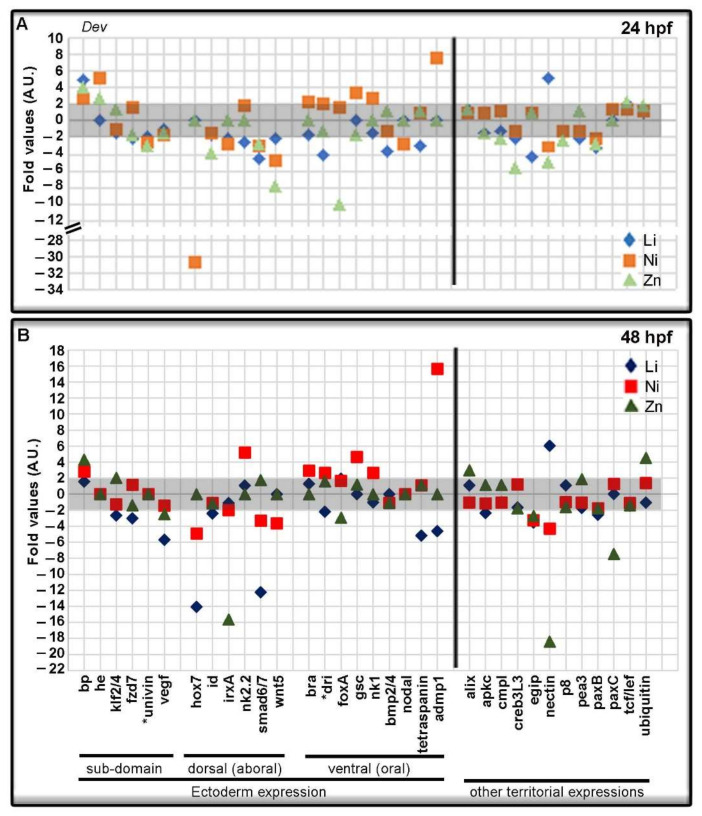
Development genes modulated by Li, Ni, and Zn treatments. The grey boxes indicate the threshold for insignificant changes following Li, Ni, and Zn treatments, i.e., fold values between −2 and + 2 at 24 (**A**) and 48 (**B**) hours post-fertilization (hpf). Symbols in the plot at 24 hpf (**A**): light blue rhombus, Li-embryos; orange square, Ni-embryos; light green triangle, Zn-embryos. Symbols in the plot at 48 hpf (**B**): blue rhombus, Li-embryos; red square Ni-embryos; green triangle, Zn-embryos. Some Development genes were organized considering their dorsal and ventral ectoderm expression. Asterisks (*) indicate genes also expressed in other territories.

**Figure 6:**
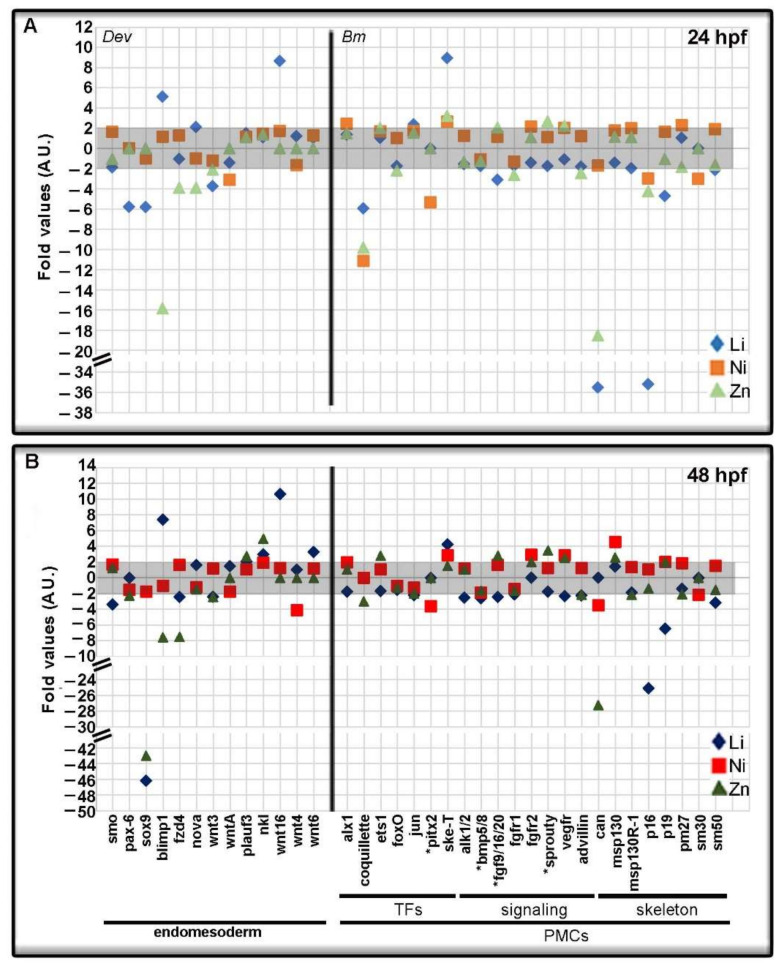
Development and Biomineralization genes with endomesoderm and mesoderm (primary mesenchyme cells, PMCs) expression modulated by Li, Ni, and Zn treatments. The grey boxes indicate the threshold for insignificant changes following Li, Ni, and Zn treatments, i.e., fold values between −2 and + 2, at 24 (**A**) and 48 (**B**) hours post-fertilization (hpf). Symbols in the plot at 24 hpf (**A**): light blue rhombus, Li-embryos; orange square, Ni-embryos; light green triangle, Zn-embryos. Symbols in the plot at 48 hpf (**B**): blue rhombus, Li-embryos; red square Ni-embryos; green triangle, Zn-embryos. The Development genes here considered have endomesoderm expression. Biomineralization genes, mainly expressed by the PMCs, were organized taking into consideration their functions, i.e., transcription factors (TFs), signaling molecules (signaling), and skeleton structures (skeleton). Asterisks (*) indicate genes also expressed in other territories.

We further summarized nCounter NanoString data in the scatter plots of Figure 4, Figure 5 and Figure 6, to analyze in detail the various genes divided into categories and sub-categories. 

In Figure 4, the data for the genes belonging to *Ap*, *Def*, *Imm*, and *Ner* categories at 24 hpf (Figure 4A) and 48 hpf (Figure 4B) are reported. Focusing on the *Ap* category, Ni did not affect genes at both endpoints analyzed, differently from Li and Zn which modulated the expression of these genes. In particular, *apaf1* was downregulated by Zn at 24 hpf (−3.16-fold) and Li at 48 hpf (−2.94-fold), while *bax* was only upregulated by Zn at 48 hpf (2,68-fold). Li upregulated *caspase-8* (3.20 and 2.70-fold) at both times, while Zn upregulated it at 48 hpf (2.51-fold). *p63* gene was upregulated at both 24 and 48 hpf by Li (2.89 and 2.44-fold) and Zn (2.52 and 6.36-fold). Among *Def* genes at both analyzed endpoints, *14-3-3epsilon* gene was downregulated by Li, Ni, and Zn with the greatest effect due to Zn treatment at 48 hpf (−9.16-fold). 

Ni affected few *Def* genes as *mt* (−2.54-fold) at 24 hpf and *hsp70-II* (2.09-fold) at 48 hpf. At 24 hpf, Li induced the downregulation of *gp96* (−2.52-fold), *mt* (−4.37-fold), and *mdrp1* (−2.42-fold) genes and the upregulation of *hsp70-II* (2.38-fold). At 48 hpf, Li affected the same genes modulated at 24 hpf with the exclusion of *gp96*, which was unaffected, and *hsp60*, which was upregulated (2.77-fold). Opposite to Li and Ni, Zn caused a general upregulation with high fold values for *hsp60* (4.58) and *hsp70-II* (16.44) at 24 and 48 hpf, while it had no effects on the *mt* gene. In addition, Zn upregulated *gp96* (2.54-fold) and faintly *p38MAPk* (2.05-fold) genes. It should be noted that the *hsp70-II* gene was significantly upregulated at 48 hpf by both Li and Zn (9.66 and 16.44-fold, respectively). 

In the *Imm* category (Figure 4), Li mainly upregulated the analyzed genes, as opposed to Zn which mainly downregulated them, while Ni affected only a few of them. The *gal-8* gene was the only gene downregulated by Li, in this category, at 48 hpf (−2.59-fold), while it was downregulated at both times (−4.78 and −6.68-fold, respectively) by Zn. *Gata1/2/3* gene was upregulated by Li (3.12 and 4.67-fold) and downregulated by Ni (−2.94 and −2.33-fold) and Zn (−2.94 and −3.43-fold) at both 24 and 48 hpf. The *gcm* gene was upregulated by Li at 24 hpf (2.13-fold), as well as *nfkb* (2.51-fold). The *pks1* gene was greatly downregulated by Ni at 24 hpf (−30.28 fold) and by Zn at both endpoints (−59.40 and −7.64-fold). 

In the *Ner* category, Li mainly caused the downregulation of nearly all the analyzed genes, with *wnt1* being the only upregulated one (4.17-fold) at 48 hpf. Ni upregulated *chordin* (2.15 and 5.83-fold), *otp* (2.39 and 3.71-fold), and *wnt1* (2.19 and 2.73) genes at both times, while it downregulated *paxpax2/5/8* (−2.65-fold) only at 24 hpf. Zn downregulated *chordin* (−3.84-fold), *pax2/5/8* (−2.97-fold), *slc6a4*/*sert* (−2.01) at 24 hpf, and *onecut/hnf6* (−3.97 and −2.03-fold) and *gfi1* (−4.99 and −2.95-fold) at both endpoints. The upregulated genes by Zn were *notch* (3.97-fold) at 48 hpf and *fzd5/8* (2.62 and 6.35-fold) at both endpoints (Figure 4).

For greater clarity in the presentation of the data, the genes of the *Dev* category have been divided into sub-categories, taking into account their spatial expression, where known, and are shown in Figure 5 and Figure 6. Figure 5 shows genes of the *Dev* category with ectodermic expression, which were modulated by the metal exposures at 24 hpf (Figure 5A) and 48 hpf (Figure 5B), respectively. Some genes, normally expressed in the specific sub-domain of the ectoderm were downregulated as *fzd7* by Li at both times (−2.03 and −3.01-fold), *univin* by the tree metals at 24 hpf (−2.00, −2.56, −2.99-fold) and *vegf* by Li and Zn at 48 hpf (−5.69 and −2.51-fold).

Many of the genes with dorsal ectoderm expression were downregulated, for example *hox7* in Ni-embryos at both times (−30.67 and −4.91-fold) and in Li-embryos at 48 hpf (−14.07-fold), *smad6/7* in all treated embryos at 24 and 48 hpf, except in Zn-embryos at 48 hpf, and *wnt5* in all treated embryos at 24 and 48 hpf, except in Li- and Zn-embryos at 48 hpf in which it was not quantifiable. The only upregulated dorsal ectoderm gene was *nk2.2* (5.19-fold) by Ni at 48 hpf. Looking at the genes with ventral expression, some of these were downregulated by Li as *bmp2/4* gene, (−3.75-fold at 24hpf), *dri* (−4.11 and −2.19-fold), and *tetraspanin* (−2.96 and −5.18 fold) at 24 and 48 hpf. Ni upregulated *brachyury* (*bra*, 2.31 and 2.93-fold), *deadringer* (*dri,* 2.07 and 2.63-fold), *goosecoid* (*gsc*, 3.44 and 4.63-fold), and *nk1* (2.65 and 2.66-fold) at both endpoints, with the exclusion of *nodal* that was downregulated (−2.85 fold at 24 hpf). At both endpoints, the only affected ventral ectodermic gene by Zn treatment was *foxA* (−10.07 and −2.91 fold). Among the other genes of the *Dev* category, those mainly affected were *admp1*, upregulated by Ni at both endpoints (7.59 and 15.66-fold) and downregulated by Li at 48 hpf (−4.59 fold); *nectin*, upregulated by Li (5.18 and 6.04 fold) and downregulated by Ni (-3.11 and 4.34 fold) and Zn (−4.92 and −18.41 fold) at both endpoints; *paxB*, downregulated by the tree agents at 24 hpf and only by Li at 48 hpf (−2.58 fold) and *paxC*, only downregulated by Zn at 48 hpf (−7.48 fold).

In Figure 6, genes belonging to the *Dev* and *Bm* categories, having endomesoderm and mesoderm expression respectively, are reported, at 24 (Figure 6A) and 48 hpf (Figure 6B). Within the *Dev* category, the transcripts expressed in mesodermic cells were downregulated, such as *smoothened* (*smo*, −3.39-fold by Li at 48 hpf), *pax-6* (−5.81-fold in Li-embryos at 24 hpf; −2.28-fold in Zn-embryos at 48 hpf) and *sox9* (−5.39 and −46.17-fold by Li at both endpoints; −42.99-fold by Zn at 48 hpf).

Looking at the genes expressed in the endoderm, the transcription factor (TF) *blimp1* was upregulated by Li (5.11 and 7.37-fold) and downregulated by Zn treatment (−15.85 and −7.58-fold) at both endpoints.

Li also upregulated *nova* (2.10-fold) at 24 hpf, *wnt16* (8.65 and 10.62-fold) at both endpoints, and *wnt6* (3.26-fold) at 48 hpf. Zn downregulated *nova* (−3.93-fold) at 24 hpf, *fzd4* (−3.92 and −7.49-fold), and *wnt3* (−2.11 and −2.45-fold) at both endpoints, the latter being the only *wnt* gene downregulated by Li at both 24 and 48 hpf. Ni only downregulated *wntA* (−3.13-fold) at 24 hpf and *wnt4* (−4.15-fold) at 48 hpf.

Among the *Bm* genes, we considered a group of TFs expressed in the skeletogenic cells, the primary mesenchyme cells (PMCs). At 24 hpf, the most affected TFs were *coquillette* and *ske-t*, which were down- and upregulated by the three metal treatments, respectively (Figure 6A), while at 48 hpf *coquillette* was downregulated by Zn (−2.98) and *ske-t* was upregulated by Li and Ni (4.26 and 2.84-fold) (Figure 6B).

Ni downregulated *pitx2* at both endpoints (−5.38 and −3.60-fold). Li had opposite effects on the *jun* gene that was upregulated at 24 hpf (2.38-fold) and downregulated at 48 hpf (−2.23-fold). Looking at the genes coding for signaling proteins expressed by PMCs, they were moderately affected by Li and Ni treatments at 24 hpf. Indeed, at both endpoints, Li downregulated (−3.09 and −2.47-fold) and Zn upregulated (2.07 and 2.79-fold) *fgf9/16/20*, while Ni upregulated *fgfr2* (2.19 and 2.90-fold), and *vegfr* (2.04 and 2.88-fold), the latter also upregulated by Zn (2.28 and 2.05-fold) (Figure 6A). Zn affected *fgfr1* (−2.61-fold) at 24 hpf and *sprouty* (2.69 and 3.45-fold) at both endpoints. At 48 hpf, Li affected other signaling genes, downregulating *alk1/2* (−2.54-fold), *bmp5/8* (−2.61-fold), *fgfr1* (−2.11-fold), and *vegfr* (−2.32-fold) (Figure 6B). 

Most of the genes whose products were directly involved in the biomineralization process were mainly downregulated by the three metals (Figure 6). *Carbonic anhydrase* (*can*), coding for an enzyme involved in the skeleton deposition and development, was greatly downregulated by Li (−32.52-fold) at 24 hpf, by Ni (−3.52-fold) at 48 hpf and by Zn (−18.49 and −27.24-fold) at both endpoints. In addition, Li downregulated three genes coding for structural proteins of the skeleton, as *p16* (−32.50 and −25.08-fold), *p19* (−4.72 and −6.49-fold) and *sm50* (−2.15 and −3.19-fold), at both endpoints. Ni downregulated *p16* (−2.95-fold) at 24 hpf and *sm30* (−3.01 and −2.17-fold) at both endpoints. The *msp130* gene, coding for a glycoprotein localized only on skeletogenic cell membranes, was upregulated by Zn (2.60-fold) and Ni (4.57-fold) at 48 hpf. A family member, *msp130R-1*, was downregulated only by Zn at 48 hpf (−2.16-fold). Ni, at 24 hpf, and Zn, at 48 hpf, affected *pm27*, coding for a structural protein, with opposite effects (2.31 and -2.08-fold, respectively).

**Figure 7:**
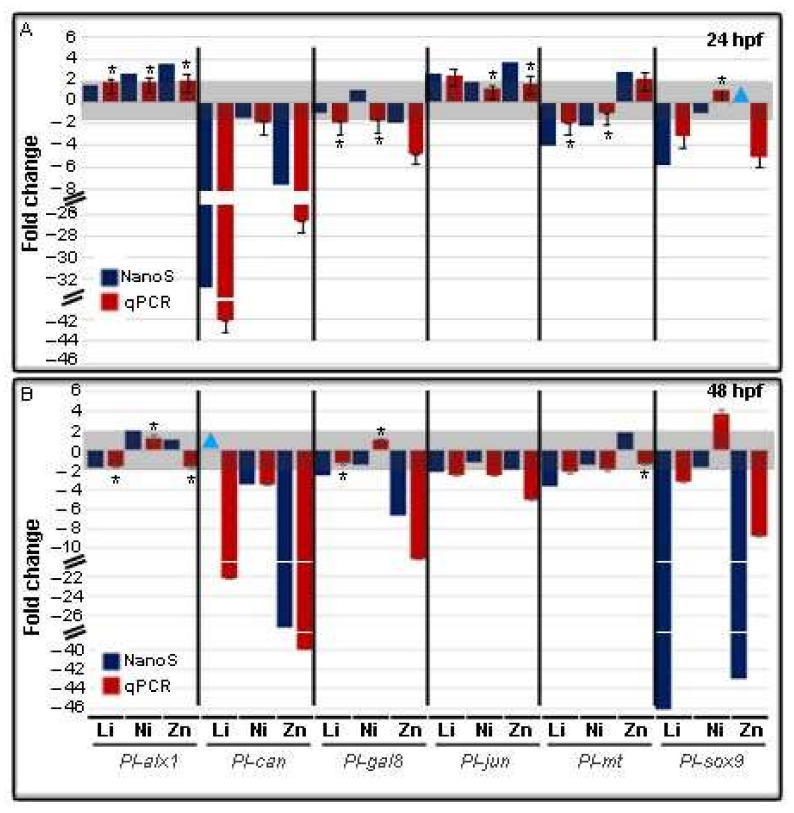
Comparison of qPCR data of gene transcription levels in Li-, Ni-, and Zn-embryos with NanoString data. *Pl-alx1, Pl-can, Pl-gal8, Pl-jun, Pl-mt,* and *Pl-sox9* mRNA levels were analyzed compared to control embryos using the endogenous gene *Pl-Z12-1* for normalization. The gray boxes indicate the threshold for insignificant changes following Li, Ni, and Zn treatments, i.e., fold values between −2 and + 2, at 24 (**A**) and 48 (**B**) hours post-fertilization (hpf). Symbols: NanoS, NanoString data, blue bars; qPCR data, red bars; Li, Li-embryos; Ni, Ni-embryos, Zn, Zn-embryos. The light blue triangles indicate not quantifiable values. Each bar of qPCR data represents the mean of three independent experiments of qPCR ± SD. QPCR mean values were significantly different according to the one-way ANOVA (*p* < 0.05), followed by the Tukey’s test. The asterisks (*) indicate statistically not significant variations to the relative control.

Among the 127 genes analyzed by nCounter NanoString methodology, we selected six target genes, representing the categories that most interested us, namely *Pl-alx1* (TF within *Bm*), *Pl-can* (skeleton within *Bm*), *Pl-gal-8* (*Imm*), *Pl-jun* (TF within *Bm*), *Pl-mt* (*Def*), *Pl-sox9* (*Dev*), and performed comparative qPCR analyses to compare the results obtained with NanoString analysis (Figure 7). 

QPCR is an alternative sensible technology to measure changes in mRNA expression levels and, also in this case, we considered significant values of fold change those higher or lower than ±2, compared to controls. In this range, the qPCR data are in agreement with those of nCounter NanoString analysis, except for a slight difference at 48 hpf in the trend of the genes *Pl-alx1* and *Pl-mt* in Zn-embryos, *Pl-gal-8* in Ni-embryos, although such variations are to be considered not significant as they remain within the threshold ±2 (Figure 7B). As for the other samples, the trend of fold changes was in good agreement between the two analyses, although the magnitude of values was sometimes quite different, as the great difference observed for *Pl-sox9* in Li- and Zn-embryos at 48 hpf. However, these differences are probably due to the diverse technologies used for the analysis, in agreement with what was reported by Prokopec et al. [36].

### 3.3. Metal-Dependent Sensitive Genes as Potential Biomarkers

**Figure 8:**
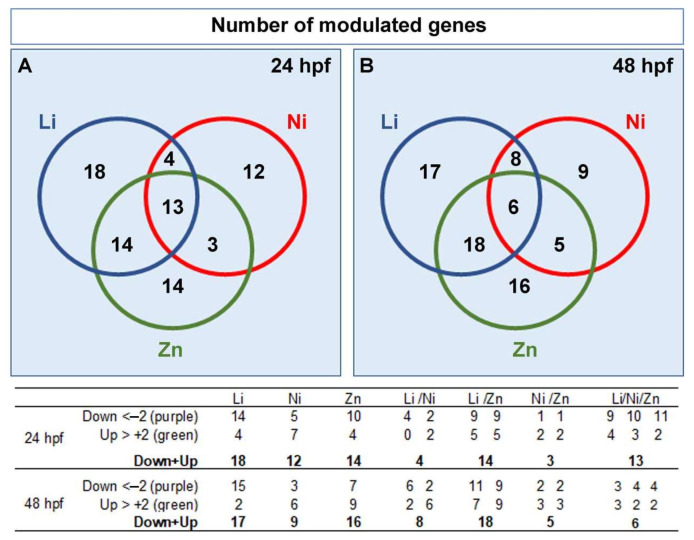
Venn diagrams reporting the numbers of modulated genes in different subgroups, i.e., in Li, Ni, Zn, Li/Ni, Li/Zn, Ni/Zn, and Li/Ni/Zn. Modulated genes at 24 (**A**) and 48 (**B**) hours post-fertilization (hpf). The table below reports the numbers of down- and up-regulated genes in each group.

Through our deep analysis, we might identify specific responsive genes for each metal, grouping those genes that are particularly sensitive to only one of the three metals analyzed, or sensitive to more than one of the metals, to be used as analytical tools for future environmental toxicity studies. The Venn diagram, and the relative table below the diagram (Figure 8), allowed to represent the amount of overlapping modulated genes and those not shared by the three metals, the latter being 18 and 17 for Li, 12 and 9 for Ni, 14 and 16 for Zn, at 24, and 48 hpf, respectively. In the Supplementary Table S2, we reported the lists of these genes, i.e., those specifically modulated by each of the three metals, those in common between Li and Ni (Li/Ni), Li and Zn (Li/Zn) or Ni and Zn (Ni/Zn) and those in common among all the three metals (Li/Ni/Zn), at 24 and 48 hpf. 

In Li-embryos at least one gene for each category was sensitive to it at 24 hpf, and many were highly modulated (8 out 18 decreased/increased more than 3-fold). Therefore, in addition to *apaf1* (*Ap*), *mt* (*Def*), and *pax2/5/8* (*Ner*), it would be possible to choose among *apkc*, *fzd1/2/7, id*, *paxB*, *smo*, *tetraspanin*, *wnt16 and wnt6* of the *Dev* category and *alk1/2*, *bmp5/8*, *fgfr1*, *jun*, *p16* and *sm50* of the *Bm* category, all genes that were not modulated by the other two metals. A similar approach could be followed to identify Zn and Ni responsive genes. In particular, genes belonging to five out six categories were specifically modulated by Zn (5 out 14 and 4 out 16 decreased/increased more than 3-fold at 24 and 48 hpf, respectively), whereas Ni modulated mainly genes belonging to *Ner*, *Dev,* and *Bm* categories (6 out 12 and 6 out 9 decreased/increased more than 3-fold at 24 and 48 hpf, respectively). 

With the aim of selecting new biomarkers, we chose to consider those genes that appeared modulated by all the three metals, particularly 13 in embryos at 24 hpf and 6 in embryos at 48 hpf (Table S2). Only three genes were modulated by all the three metals at both 24 and 48 hpf, i.e., *14-3-3 epsilon, gata1/2/3,* and *nectin*. Others were downregulated (*pax2/5/8*, *paxB*, *smad6/7*, *univin, wnt5, p16,* and *coquillette*) or upregulated (*bp10* and *ske-t*) by all three metals only at 24 hpf or only at 48 hpf, i.e., *hsp70-II*, *egip precursor* and *vegfr*. 

### 3.4. Metal-Dependent Sensitive Genes and Functional Enrichment

Metal-related impacts on gene expression were seen in all the treatments with some overlapping in the enrichment of various KEGG Orthology (KO) pathways. Table S3 lists the *S. purpuratus*-specific versions of KEGG pathways and/or BRITE hierarchies found for the three metals.

The KO analysis showed that most of the genes modulated by Li were related to signal transduction pathways [09132], in particular, Wnt signaling pathway [PATH:spu04310], TGF-beta signaling pathway [PATH:spu04350], notch signaling pathway [PATH:spu04330], foxO signaling pathway [PATH:spu04068], and to transcription factors [BR:spu03000], in addition to endocytosis [PATH:spu04144], G protein-coupled receptors [BR:spu04030] and peptidases and inhibitors [BR:spu01002], at both endpoints (Table S3). Zn modulated genes related to Wnt signaling pathway [PATH:spu04310], notch signaling pathway [PATH:spu04330] and foxO signaling pathway [PATH:spu04068], in addition to protein processing in endoplasmic reticulum [PATH:spu04141], ubiquitin-mediated proteolysis [PATH:spu04120], membrane trafficking [BR:spu04131], cytoskeleton proteins [BR:spu04812] and transcription factors [BR:spu03000], mainly at 48 hpf (Table S3). At 24 hpf, Ni modulated genes related to TGF-beta signaling pathway [PATH:spu04350] and transcription factors [BR:spu03000], whereas only genes related to Wnt signaling pathway [PATH:spu04310] were modulated at 48 hpf (Table S3). Enrichment of DNA repair and recombinant proteins [BR:spu03400] was found for all three metals.

## 4. Discussion

In this study, we report an in-depth characterization of the response at the molecular level to Li, Ni, and Zn of the *P. lividus* sea urchin embryo, through a high-throughput screening approach using the nCounter NanoString technology. We have analyzed 127 genes selected from the NCBI database and arbitrarily organized them into six different functional categories based on an in-depth study of the data in the literature. As a whole, the modulation of the expression of the various genes represents a synthesis of the molecular defense systems adopted by *P. lividus* embryos to counteract Li, Ni, and Zn effects, respectively. The activation of the defense systems involves an energetic cost for the embryos, which results in developmental alterations and the formation of alternative morphotypes. Furthermore, we need to take into consideration the dual role of some of the defensive genes and proteins, i.e., in the regulation of developmental processes and in the protection against environmental risks, so that their recruitment into protective mechanisms might affect embryonic development. 

We focused our study on a short period of time, i.e., 0−48 h of sea urchin embryo development, to evaluate the effects of Li, Ni, and Zn treatments at sublethal concentrations, not environmentally relevant, but capable of producing homogeneous populations of abnormal embryos, each with its own characteristic morphology. Indeed, various morphological malformations have been already described in different sea urchin species treated with Li, Ni, and Zn [20,21,37,38], including our recent study on *P. lividus* [26]. The most striking morphological effects of Li, Zn, and Ni treatments are on the axes: the animal/vegetal (A/V) one in vegetalized Li-embryos, which have less ectoderm and more endomesoderm than animalized Zn-embryos that, on the contrary, have more ectoderm and less endomesoderm, while Ni treatment affects the establishment of dorso/ventral (D/V), also called oral/aboral (O/A), axis. While the A/V axis is already present in the egg and it is rigidly fixed, the D/V axis is established after fertilization through complex molecular events, recently reviewed by Molina and Lepage [39], and is well known to be easily disturbed by many physical or chemical treatments and agents.

In addition to perturbing the axes patterning, the embryonic nervous system appeared to be affected mainly by Zn, but also somewhat by Li and Ni, on the basis of the embryonic localization of two neural markers, Sox2, and ciliary band. Firstly, a slight difference in the localization of Sox2 in *P. lividus* compared to *S. purpuratus* embryos has to be highlighted. In particular, the *soxB2* gene, homolog to the human *sox2*, is expressed in the animal pole domain (which contains neurogenic ectoderm) of early *S. purpuratus* gastrula at 30 hpf and in the oral ectoderm around the ciliary band in pluteus at 72 hpf, while no signal was detected in the aboral ectoderm of pluteus [40]. Partially in agreement, the anti-Sox2 antibody labeled mainly the ciliary band in *P. lividus* pluteus at 48 hpf, but the protein was also present in some cells of the aboral ectoderm of the pluteus apex, which can therefore be neurogenic cells too. The sox2-expressing cells were lost in nearly all metal-treated embryos, in addition to the two small central ganglionic structures labeled by Ab-295. These results reveal that Li, Ni, and Zn might alter the embryonic nervous system, in agreement with data obtained from the Nanostring analysis as discussed in the following. 

### 4.1. Looking for Potential Biomarkers

The morphological abnormalities of *P. lividus* embryos treated with metals are obviously associated with changes in gene expression and, consequently, in signaling pathways. Indeed, several genes appeared modulated by Li, Ni, and Zn at 24 (49, 33, and 44 out of 127, respectively) and 48 hpf (49, 29, and 45 out of 127, respectively), of which 13 and 6 were the same for the three treatments (shown in the Venn diagram of Figure 8). In Table 1, we listed the genes affected by Li, Ni, and Zn that can be used as potential biomarkers for all of them. In the following, we will provide some information on each of these genes, when available, to highlight the molecular mechanism of the stress response of sea urchins exposed to Li, Ni, and Zn.

Three genes were affected by all three metals at both endpoints, 24 and 48 hpf, i.e., *14-3-3epsilon*, *gata1/2/3*, and *nectin*. The *14-3-3epsilon* belongs to a highly conserved family of eukaryotic adaptor proteins found in diverse organisms and involved in many cellular processes. For example, 14-3-3 proteins interact with bax preventing its translocation into mitochondria and playing an anti-apoptotic role [41]. In sea urchin embryos, increased *Pl-14-3-3epsilon* mRNA levels were found following UVB irradiation [30], suggesting its implication in the regulative cascade activated in the stress response.

The *gata1/2/3* gene codes for a DNA binding zinc finger TF that is involved in vertebrate hematopoiesis. Regarding its sea urchin homologs, *Spgatac* regulates immune cell specification in embryos and larvae [42], while *Spgatae* might be a useful biomarker for assessing the sea urchin hypoxic response, as suggested by its downregulation in the adult immune cells of *S. nudus* maintained in hypoxic condition [43]. 

*Nectin* is one of the proteins constituting the extracellular matrix (*ECM*) of the sea urchin embryo, known to be involved in the ecto-mesoderm signaling regulating skeleton development [44]. The changes in *nectin* gene expression are in good agreement with the defects in the development of the skeleton observed in all metals-treated embryos, so much so that it can be taken into account among the potential biomarkers. 

Among the genes affected by the three metals only at 24 hpf, *chordin* and *pax2/5/8* [45] belong to the Ner category, interesting for new studies of the nervous responses to the toxic action of pollutants. A recent review well described the embryonic neurogenesis in echinoderms, which proved to be a very complex process, and the molecular toolkit involved [35], which can therefore be considered in the search for new biomarkers of the stress response. 

In the *Dev* category, six genes were modulated by the three metals at 24 hpf. Although little is known about the *blastula protease* (*bp*) 10 gene [46] and its ectoderm expression, we consider it rather interesting as *bp10* expression was modulated also by Cd treatment [25], in addition to Li, Ni, and Zn. 

The *paxB* gene belongs to the PAX family of transcription factors, which have been characterized in sea urchin embryos and are reported to be involved in visual system development in a variety of species [47].

Four genes constitute the sea urchin repertoire of *smad* genes that are a family of TFs activated downstream the TGF-beta signaling [48] and, among these, *smad6* is known to be expressed by the presumptive dorsal ectoderm [49].

*Univin* is a growth factor of the TGF-beta superfamily involved in sea urchin skeleton morphogenesis [50]. It could be an interesting biomarker of different pollutants, for example, its downregulation was observed in *P. lividus* embryos exposed to gadolinium (20 μM) for 48 h [51].

*Wnt5* acts with a short-range signal that activates a narrow band of ectodermal cells from the endoderm, integrating different types of positional information to correctly locate the production site of signals needed to guide skeletogenesis [52].

Certainly, the biomineralization genes would be interesting molecular markers, to put beside the morphological observation of the absence of the skeleton in embryos exposed to the three metals. In this respect, *p16*, coding for an acidic phosphoprotein of the skeleton [28], as well as *ske-t* and *coquillette*, two TFs belonging to the T-box family, should thus be taken into consideration. *Ske-T* is expressed early in the *S. purpuratus* embryos [53]. *Coquillette*, a member of the Tbx2 subfamily, is expressed late in the *S. purpuratus* embryo, restricted to the PMCs of gastrula embryos, involved in the skeleton formation [54].

Only six were the genes affected by all three metals at 48 hpf. Within the *Def* category, in addition to the *14-3-3epsilon* gene previously described, members of the Hsp family, such as *hsp70-II*, were expected to be modulated. The upregulation of *hsp* genes is a typical stress response, well known in many organisms, which can have an anti-apoptotic effect [55], as also suggested for sea urchins [56].

The *exogastrula-inducing peptides* (*egip*) gene codes for small epidermal growth factor-related peptides, which probably are secreted and then associated with the ECM, although their role is not yet known [57].

*Vegfr* is involved in the VEGF signaling that guides skeleton morphogenesis from the ectoderm [58] and it is one of the genes grouped in the *Bm* category. Most of these genes are involved in embryonic skeleton development and are mainly expressed by the PMCs, clearly recognizable in Li, Ni, and Zn embryos, even if not properly organized [26]. Together with other genes of the *Bm* category, this gene seems an interesting potential molecular biomarker, since its modulation could be associated with morphological observations on the skeleton development. 

Beyond the potential biomarkers proposed in Table S2, some other genes could be considered potential biomarkers, both because they are greatly affected by one or more of these metals and because they can have interesting functions in the sea urchin. In particular, we could suggest *can*, *hox7*, *foxA*, *pks1*, *irxa*, *sox9*, and *wnt16*.

*Can* codes for a carbonic anhydrase metalloenzyme, which has a key role in the biomineralization process in many metazoans, also including sea urchins [59]. Interestingly, its expression is often affected by negative external stimuli, such as it is strongly downregulated by Cd and Cd in combination with UVB radiation in *P. lividus* embryos [25]. 

Within the *Imm* category, the *pks1* gene, found in many organisms, is greatly affected by Ni and Zn in agreement with studies reporting its reduced expression by Ni in *L. variegatus* [60] and in *P. lividus* embryos [33]. In sea urchins, the PKS enzyme is involved in the synthesis of the red pigment echinochrome A and is expressed by pigment cells, a type of embryonic immune cell [61]. The modulation of *pks1* can be associated with the morphological observation, checking the presence/absence of pigment in exposed embryos. For example, the absence of pigment cells was observed in *P. lividus* Ni- and Zn-embryos (this study; [26]) and in *L. variegatus* Ni-embryos [21]. Growing evidence supports the importance and role of metal ions in the immune system, both regulating the innate immunity and host defense functions, in what is now called “metal-controlled immunity” [62]. In this regard, the studies on the immune response induced by metals in a simple model organism such as the sea urchin might be of great support.

*Sox9* belongs to the *Dev* category and, more in detail, to the gene subgroup having endo-mesoderm territorial localization. Interestingly, other agents, such as UVB and Cd, caused its downregulation in *P. lividus* embryos [25]. *Sox9* gene, also known as *Sp-soxE* in the *S. purpuratus* genome (www.echinobase.org (accessed on 1 January 2019)) [63], belongs to a family of minor groove DNA binders, widely expressed during sea urchin embryo development, and is one of the regulatory genes expressed during embryonic myogenesis [64].

*Irxa* and *hox7* are two dorsal genes [49] grouped in the *Dev* category, together with many genes involved in axes specification and patterning, including A/V and D/V, which were differently altered by the three metals, as described above. 

The *foxa* gene, a forkhead transcription factor, is an integral component of the endoderm specification subcircuit of the endomesoderm gene regulatory network, as characterized in the *S. purpuratus* embryo [65]. The Authors showed that blocking *foxa* expression, by the injection of morpholino antisense oligonucleotides at high concentrations, caused the failure of gut formation, a process that involves the regulation of structural genes and cell adhesion molecules [65]. The *foxa* gene, and the morphology of the embryonic gut, could be interesting to consider as pollutant biomarkers.

Most of the Wnt and their receptor frizzled (*fzd*) genes have expression domains associated with archenteron development, thus it was not surprising that *wnt16* was greatly upregulated by Li. Specifically, at the late gastrula stages, different populations of endodermal cells expressed *wnt16*. At later stages, prism and early pluteus, the expression of *wnt16*, as many other Wnt genes, becomes even more restricted in a full ring around the anus [66].

Altogether these results provide a general framework for the possible responses at the molecular level of sea urchin embryos to different metals, from which to extrapolate useful information on possible new markers to be taken into account in ecotoxicological studies. Indeed, the reorganization of the embryonic territories induced by the different metals, clearly distinguishable at the morphological level, i.e., animalization (Li), vegetalization (Zn), and radialization (Ni), could now be associated with some genes modulated exclusively by each of the three metals. Nevertheless, among the various aspects that must be considered in the evaluation of metals toxicity, i.e., their concentration, the different sensitivities of the embryonic stages, and of the various species of sea urchins, it would be worthwhile to identify a panel of modulated genes for each metal.

### 4.2. Analysis of Pathways Affected by Metals

The data obtained by the analysis of pathways enrichment can provide further information on the molecular responses of sea urchin embryos exposed to Li, Ni, and Zn, useful for future exploitations in ecotoxicological studies. 

Only 39 of the 82 genes modulated by each metal and those modulated by all the three metals were found in 13 pathways by KEGG Orthology (KO, https://www.genome.jp/kegg/ko.html (accessed on 1 June 2022)) analysis (Table S3). The pathway of TFs was the most enriched, followed by the signaling pathways of Wnt, TGF-beta, FoxO, and notch, as well as the pathways of protein processing in the endoplasmic reticulum, endocytosis, membrane trafficking, cytoskeleton proteins, G protein-coupled receptors, DNA repair and recombinant proteins, peptidases and inhibitors, and ubiquitin-mediated proteolysis. All the three metals impacted the TFs pathway, modulating 11 genes involved in numerous and different cellular processes. 

It seems evident that the main enriched pathways found by the KO analysis were the signaling ones, which control all aspects of embryo development. In particular, all the metal treatments here analyzed impacted the Wnt signaling pathway, up or downregulating some of the related genes (*fzd1/2/7*, *fzd4*, *wnt16, wnt6*, *wnt4*, *wnt5*, *tcf/lef*). The Wnt signaling pathway is highly evolutionarily conserved in organisms, as it is involved in body axis patterning, cell proliferation and migration, and cell fate specification. In sea urchins, this signaling pathway has been extensively studied and is known to be a crucial regulator of embryo development [67], including the A/V axis formation. Furthermore, it is known to be involved in neurogenesis, restricting neuroectoderm to the anterior pole of the embryo with precise spatio-temporal control, involving also JNK signaling, in addition to *wnt1* and *wnt8* [68]. The main receptors for Wnts are Frizzled receptors that are involved in the formation and function of the neuronal circuits in mammalian central nervous system [69]. 

The third most enriched pathway for all metals was the TGF-beta signaling (*bmp2/4*, *nodal*, *pitx2*, *chordin*, *smad6/7*), involved in a wide range of cellular processes and highly conserved all over the animal world. In addition to ligands and receptors, the TGF-beta signaling pathway requires the involvement of receptor-regulated proteins (the intracellular mediators Smads) and various TFs to regulate the expression of target genes. Among the genes belonging to this KO pathway, *chordin* was included in our *Ner* category, as it is one of the marker genes for apical and neural territories, in addition to *gfi1* [49], *onecut/hnf6* [70], *pax6*, *sox9*, and *pax2/5/8*. 

Under Li and Zn treatments, but not Ni, some pathways were enriched, for example FoxO signaling pathway (Li, Zn), notch signaling pathway (Li, Zn), endocytosis (Li, Zn), G protein-coupled receptors (Li), peptidases and inhibitors (Li), membrane trafficking (Zn), cytoskeleton proteins (Zn), and ubiquitin-mediated proteolysis (Zn).

Most interesting for our study, are the three genes (*foxG*, *foxO*, *p38MAPK*) that enriched the FoxO signaling pathway. The Forkhead (Fox) family includes TFs regulating target genes involved in diverse biological processes, proliferation, differentiation, longevity, metabolism, and apoptosis. In the sea urchin, at least 22 genes belonging to the FOX family have been annotated [71], although many of them have not yet been characterized. The two *fox* genes analyzed are differently expressed in late sea urchin embryos, i.e., *foxO* is expressed in PMCs, suggesting its involvement in biomineralization, whereas *foxG* expression is restricted to the ciliated band, suggesting an involvement in nervous processes [71]. By our KO analysis, *p38MAPK* gene enriched the FoxO signaling pathway, even though this gene is known to have a dual role in sea urchin, i.e., an essential regulator of embryonic development, involved in D/V axis specification and skeletogenesis [72], and a stress marker [73].

Interestingly, the functional categories of protein processing in the endoplasmic reticulum and DNA repair and recombinant proteins were impacted by the three metals, i.e., the genes *bax*, *hsp70-II*, and *14-3-3epsilon* that indeed are stress marker genes.

We believe that this study not only can greatly facilitate the identification of metal-specific biomarkers but can also be useful for the identification of functional developmental pathways altered by pollutants in *P. lividus* embryos. This is obviously a preliminary study, considering that the metals were administered individually, while in nature the effects are due to the combined action of several toxic agents.

## 5. Conclusions 

As many industrial activities release metals that can be harmful to human health as well as to the environment, it is extremely important to develop new tools that can detect these pollutants quickly and easily, but at the same time with high levels of sensitivity. In this direction, our study contributes both to expanding the knowledge of the molecular biomarkers that could be used in the environmental monitoring, and to facilitating the identification of panels of modulated genes to be used in the evaluation of metals toxicity.

The sea urchin embryo is a well-known model used in ecotoxicological studies to detect a wide range of chemical, physical and natural agents, such as metals, radiations, ocean acidifications, and sediments as well as to study the effects of combined agents. In addition, a huge amount of data is now available concerning the defensive, nervous, and immune systems and the related GRN sub-circuits governing them in sea urchin embryos, which provides new attractive objects of study, such as the immune and nervous responses to the toxic action of pollutants.

All of this can be of great help to provide new approaches to address ecotoxicological studies, encouraging new possible monitoring and intervention strategies.

## Figures and Tables

**Table 1:** List of sensitive genes common to Li, Ni and Zn as potential biomarkers of impact.

Endpoint	*Gene*	Accession Number	Category	Function
24 hpf	*14-3-3epsilon*	AJ493680.2	Defense	protein adaptor
	*gata1/2/3*	GQ377404.1	Immune	TF
	*chordin*	FJ976182.1	Nervous	extracellular modulator
	*pax2/5/8*	AF016884.1	Nervous	TF
	*bp10*	X56224.1	Development	Enzyme
	*nectin*	AJ578435.2	Development	extracellular matrix protein
	*paxB*	AF016885.1	Development	TF
	*smad6/7*	HM449802.1	Development	TF
	*univin*	DQ536195.1	Development	TGF-β ligand
	*wnt5*	HM449806.1	Development	secreted glycoprotein
	*p16*	FR693763.1	Biomineralization	spicule matrix protein
	*coquillette*	AJ508929.1	Biomineralization	TF
	*ske-t*	AJ309216.1	Biomineralization	TF
48 hpf	*14-3-3epsilon*	AJ493680.2	Defense	protein adaptor
	*hsp70-II*	X16544.1	Defense	hsp family
	*gata1/2/3*	GQ377404.1	Immune	TF
	*egip precursor*	HM449819.1	Development	Polypeptide
	*nectin*	AJ578435.2	Development	extracellular matrix protein
	*vegfr*	AM419057.1	Biomineralization	VEGF receptor

## Data Availability

Not applicable.

## Supplementary Materials

The following supporting information can be downloaded at: https://www.mdpi.com/article/10.3390/toxics10060325/s1, Table S1: Fold-change values of the analyzed genes. Table S2: Modulated genes. Table S3: KEGG pathways and/or BRITE hierarchies.
